# Wireless-Channel Key Distribution Based on Laser Synchronization

**DOI:** 10.3390/e26030181

**Published:** 2024-02-21

**Authors:** Junpei Xu, Anbang Wang, Xinhui Zhang, Laihong Mo, Yuhe Zhang, Yuehui Sun, Yuwen Qin, Yuncai Wang

**Affiliations:** 1 Institute of Advanced Photonics Technology, School of Information Engineering, Guangdong University of Technology, Guangzhou 510006, China; 2112103099@mail2.gdut.edu.cn (J.X.); abwang@gdut.edu.cn (A.W.); 2112115125@mail2.gdut.edu.cn (X.Z.); 2112103138@mail2.gdut.edu.cn (L.M.); 2112103019@mail2.gdut.edu.cn (Y.Z.); yhsun@gdut.edu.cn (Y.S.); qinyw@gdut.edu.cn (Y.Q.); 2 Key Laboratory of Photonic Technology for Integrated Sensing and Communication, Ministry of Education of China, Guangdong University of Technology, Guangzhou 510006, China; 3 Guangdong Provincial Key Laboratory of Information Photonics Technology, Guangdong University of Technology, Guangzhou 510006, China

**Keywords:** wireless security, physical layer security, key distribution, nonlinear systems, laser synchronization

## Abstract

We propose and experimentally demonstrate a wireless-channel key distribution scheme based on laser synchronization induced by a common wireless random signal. Two semiconductor lasers are synchronized under injection of the drive signal after electrical-optical conversion and emit irregular outputs that are used to generate shared keys. Our proof-of-concept experiment using a complex drive signal achieved a secure key generation rate of up to 150 Mbit/s with a bit error rate below 3.8 × 10^−3^. Numerical simulation results show that the proposed scheme has the potential to achieve a distribution distance of several hundred meters. It is believed that common-signal-induced laser synchronization paves the way for high-speed wireless physical-layer key distribution.

## 1. Introduction

With the increase in wireless communication rates, high-speed key distribution has become more important [1]. Existing key distribution is divided into two types: computational security-based and information-theoretic security-based key distribution. The former presupposes that the eavesdropper’s computational power is constrained, thus an inherent risk of being decoded by using high-performance computers. The latter has attracted increasing attention because of its ability to resist unlimited computational power. It has been proved that two users with access to common random sources can generate a shared secret key by exchanging messages over a public channel [2,3]. Many key distribution schemes based on common random sources have been proposed and investigated [4,5,6,7,8,9,10].

Information-theoretic security key distribution has been successfully demonstrated in the wireless domain [11,12,13], where the channel reciprocity properties of the wireless fading channel provide the common random sources for key establishment. However, limited by the bandwidth of channel randomness, the scheme has limited key rates (bit/s~kbit/s). Although various approaches have been investigated to improve the key rate [14,15,16], the improvement effect is quite limited. Additionally, the key generated in a static or free space environment lacks randomness, rendering it susceptible to prediction.

It has been demonstrated that optical chaos in semiconductor lasers is a promising physical entropy source for generating high-speed random bits at Gbit/s or even Tbit/s [17,18]. The synchronization of optical chaos can be used as a source of common randomness and has been intensively investigated for applications in high-speed secure key distribution [19,20,21,22,23,24]. Uchida et al. firstly experimentally demonstrated a 182-kbit/s key distribution using the chaos synchronization of two optical-feedback lasers driven by a common random light [20]. The security of the scheme is based on information theory and the practical difficulties of completely observing the fast and complex optical process [8,25]. The key rate of the scheme is limited by the chaos synchronization recovery time in the magnitude of tens of nanoseconds. Recently, Wang et al. experimentally demonstrated a 0.75-Gbit/s key distribution by using the open-loop configuration of commonly driven lasers [26]. Nevertheless, the utilization of these schemes in wireless secure communication is restricted due to the presence of optical fiber links among users.

In this paper, we propose and demonstrate a high-speed wireless key distribution scheme based on laser synchronization induced by a common wireless random signal. It enhances the key distribution scheme based on common-signal-induced synchronization to accommodate wireless environments. We successfully achieve synchronization of commonly driven lasers and demonstrate a secure key generation rate of up to 150 Mbit/s while maintaining a bit error rate (BER) below the threshold of 3.8 × 10^−3^. In addition, the distribution distance of the proposed scheme is studied by numerical simulation.

## 2. Principle and Experimental Setup

**Figure 1:**
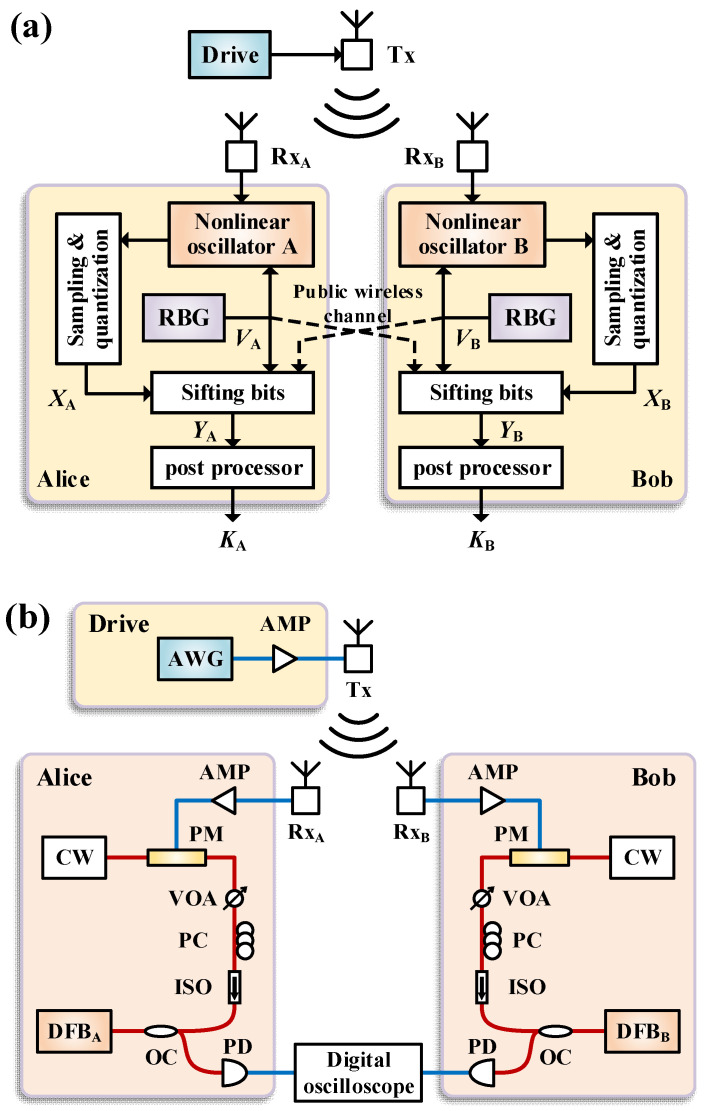
(**a**) Principle and (**b**) experimental setup for wireless-channel key distribution. RBG, random bit generator; AWG, arbitrary waveform generator; AMP, radio frequency amplifier; PM, electro-optic phase modulator; CW, continuous-wave laser; VOA, variable optical attenuator; PC, polarization controller; ISO, optical isolator; OC, optical fiber coupler; DFB, distributed-feedback semiconductor laser; PD, photodetector.

The principle of the proposed scheme is shown in Figure 1a. Two identical nonlinear oscillators are authorized to the legitimate users Alice and Bob. Each nonlinear oscillator has a set *V* of parameters that can be changed. These parameters can take on one of *M* possible values. *V_A_* and *V_B_* are generated by two independent random bit generators (RBG). A complex drive signal is transmitted through an antenna, and then it is received and injected into the nonlinear oscillators to achieve chaos synchronization. Only in the time slots when *V_A_* = *V_B_*, do the chaotic waveforms synchronize. This process is called random keying. Subsequently, the output signals of the oscillators are sampled and quantized to generate the correlated random bits *X_A_* and *X_B_* privately and independently. They repeat this procedure many times to acquire sequences of the pairs (*V_A,i_*, *X_A,i_*) and (*V_B,i_*, *X_B,i_*), *i* = 1, 2, …, *n*, respectively. Next, they exchange the parameters sequences {*V_A,i_*} and {*V_B,i_*}, *i* = 1, 2, …, *n*, over a public channel to sift bits. Only the bits *X_A,i_* and *X_B,i_* for *i* such that *V_A,i_* = *V_B,i_* are retained, otherwise the bits are discarded. These retained bits, denoted as {*Y_A,i_*} and {*Y_B,i_*}, *i* = 1, 2, …, *n*, are used to generate a common secret key *K_A_* = *K_B_* by using an information reconciliation protocol and a privacy amplification protocol [27] in post processors.

To evaluate the security of this scheme, we assume an eavesdropper, Eve, who can inject the drive signal into one or more nonlinear oscillators. Additionally, we assume that Eve can obtain any information exchanged through a public channel between Alice and Bob. It has been proven that Alice and Bob can generate a key that is completely secret to Eve when Eve has no way of inferring the bits generated by Alice and Bob without errors [8]. In our scheme, we utilize two characteristics of common-signal-induced synchronization to facilitate this condition [28,29]. Firstly, the outputs of two nonlinear oscillators can synchronize under the drive of a common noise signal, while there is no simple functional relationship between the drive signal and the oscillator outputs. This makes it difficult for Eve to obtain sufficient information about the retained bits even if she can get the drive signal from the wireless channel. Secondly, the synchronization is sensitive to the parameter mismatches of the nonlinear oscillators. This means that even if Eve can access all information on the public channel, it is difficult for Eve to obtain enough oscillators with parameter matches to observe the outputs for all possible parameter values. These characteristics give Alice and Bob a certain probability of retaining bits that cannot be obtained by Eve, thus enabling the establishment of a common secret key. It is worth noting that Alice and Bob can further reduce the amount of information obtained by Eve by applying privacy amplification techniques [27], so they do not need to know which bits Eve has obtained.

In the subsequent section, we conduct a proof-of-concept experiment using semiconductor lasers as nonlinear oscillators to verify the feasibility of the scheme and estimate the secure key generation rate under reasonable assumptions.

Figure 1b illustrates the experimental setup for wireless-channel key distribution. Alice and Bob are assigned a pair of parameter-matched distributed-feedback semiconductor lasers (DFB). These lasers were selected from an identical fabrication batch that was supplied by the company Eblana Photonics. An arbitrary waveform generator (AWG) generates a noise signal as the common wireless drive signal, which is broadcasted via an antenna (5.38~8.17 GHz standard gain horn antenna) Tx. The drive signal is transmitted over a 2.5 m wireless link and received by antennas Rx_A_ and Rx_B_. Due to the beamwidth of the transmitter, Rx_A_ and Rx_B_ are placed approximately 0.5 m apart to ensure that the drive signal is successfully received. For each user, the received RF drive signal is first converted into an optical signal using an electro-optic phase modulator (PM) of a continuous-wave laser (CW) light. The resulting phase-random drive light is then injected into the DFB laser. To adjust the modulation depth, a radio frequency amplifier (AMP) is used. The modulation depth is defined as 3*σ*/*V*_π_, where *σ* is the standard deviation of the drive signal and *V*_π_ is the half-wave voltage of the PM. For injection of the drive light, a polarization controller (PC) is used to match injection light polarization to that of the laser, and an optical isolator (ISO) is used to ensure unidirectional injection. A variable optical attenuator (VOA) is used to adjust the injection strength, which is defined as power ratio of DFB free output to drive light. The output of the laser is derived from an optical fiber coupler (OC), detected by a photodetector (PD), and observed using a digital oscilloscope (Keysight Technologies, Santa Rosa, CA, USA, DSAV164A, 16 GHz bandwidth). Although various schemes [20,26,30,31] can accomplish random keying, this does not impact the feasibility of the proposed scheme. Hence, there are no experimental demonstrations of random keying in this work, and our analysis solely focuses on its impact on the key rate.

## 3. Experimental Results

### 3.1. Laser Synchronization Induced by a Common Wireless Signal

**Figure 2:**
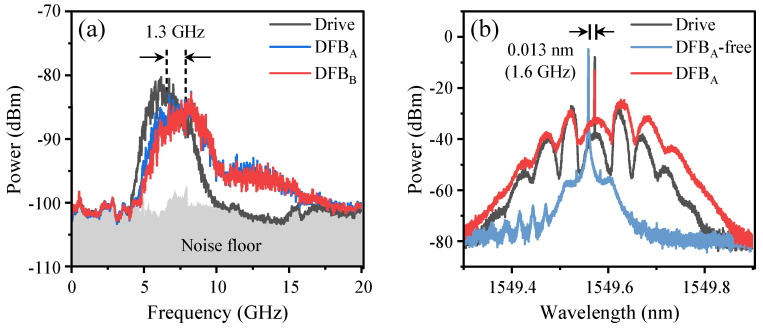
(**a**) RF spectra for the drive signal and the DFB laser outputs. (**b**) Optical spectra for the drive light and the DFB_A_ output with and without optical injection.

The drive signal utilized in our experiment is a Gaussian noise characterized by a 3-dB bandwidth of 2.8 GHz and a central frequency of 6.7 GHz. The spectrum of the drive signal is shown in Figure 2a. The generation of broad-spectrum and synchronized outputs by the lasers requires a drive signal with an adequately broad bandwidth. The power loss is 37 dB, as the received power is approximately −24 dBm and the transmit power is 13 dBm. The modulation depth is adjusted to 0.7 with an amplifier gain of 44 dB. The currents for the DFB lasers are set to 24.20 mA (2.12 *I_th_*) and 23.50 mA (2.19 *I_th_*) to achieve identical relaxation oscillation frequencies of 5.5 GHz. The injection strengths are adjusted to −8.43 dB and −8.70 dB to induce injection locking. The optical frequency detuning of the center frequencies of the DFB lasers and drive light is approximately 1.6 GHz, as illustrated in Figure 2b.

As shown in Figure 2a, the laser outputs exhibit broad spectra characterized by a 3-dB bandwidth of approximately 2 GHz. This characteristic renders them suitable for the generation of high-speed random bits. The laser outputs have center frequencies that are approximately 1.3 GHz greater than those of the drive signal. This phenomenon arises as a result of the drive signal’s spectrum encompassing the laser’s relaxation oscillation frequency, thereby generating a light injection effect that enhances the relaxation oscillation frequency [32]. The spectral characteristics of the laser output and the drive light exhibit a comparable configuration, as illustrated in Figure 2b. The laser output maintains a fixed center frequency in accordance with that of the drive light.

We introduce a measure of cross-correlation to evaluate the quality of the laser synchronization and the correlation between the laser output and the drive signal. The cross-correlation of temporal waveforms *x*(*t*) and *y*(*t*) is defined as

(1) $${CC}_{xy}=\frac{\langle \left(x\left(t\right)-\bar{x}\right)\left(y\left(t\right)-\bar{y}\right)\rangle }{\sigma_{x}\sigma_{y}},$$

where <·> denotes time average, $\bar{x}$ and $\bar{y}$ are the mean values, *σ_x_* and *σ_y_* are the standard deviations. If *CC_xy_* = 1, it indicates that *x*(*t*) and *y*(*t*) are completely identical. Conversely, if *CC_xy_* = 0, it means that they are completely uncorrelated.

**Figure 3:**
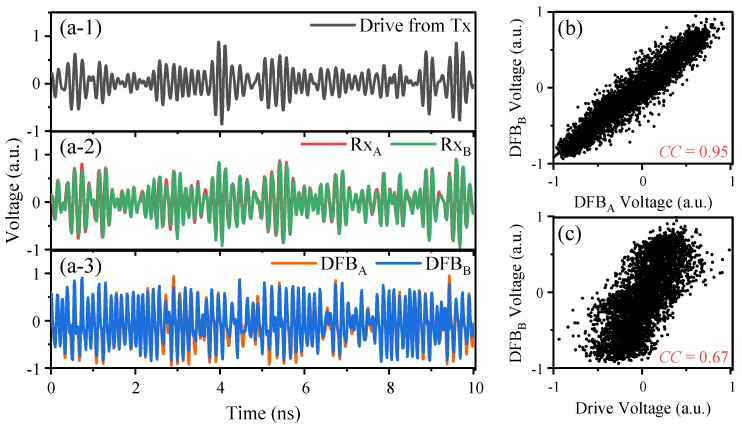
Temporal waveforms of (**a-1**) the drive signal, (**a-2**) signals received by antennas Rx_A_ and Rx_B_, and (**a-3**) the DFB laser outputs. (**b**) Correlation plots for the DFB_A_ and DFB_B_ outputs. (**c**) Correlation plots for the drive signal and DFB_B_ output.

Figure 3a shows the temporal waveforms of the transmitted wireless drive signal, the signals received by antennas Rx_A_ and Rx_B_, and the response outputs of the DFB lasers. The received signals of both users maintain a high similarity, which satisfies the conditions for common-signal-induced synchronization. The temporal waveforms produced by the lasers demonstrate a significant resemblance. The corresponding linear correlation shown in Figure 3b is also evident in their corresponding correlation plot, which has a cross-correlation of 0.95. This observation demonstrates that Alice and Bob have successfully executed high-quality chaotic synchronization. In addition, as shown in Figure 3c, the cross-correlation between the laser output and the drive signal is 0.67, which is comparatively lower than that between the laser outputs. This indicates that only limited information about the synchronized outputs can be obtained from the drive signal, which satisfies our condition for key distribution.

**Figure 4:**
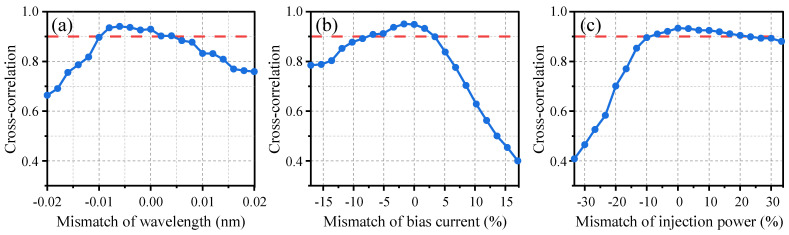
Effects of external parameters mismatch on laser synchronization: (**a**) center wavelength mismatch, (**b**) bias current mismatch, and (**c**) injection power mismatch. Original settings: center wavelength *λ_A_* = *λ_B_* = 1549.559 nm, bias current *I_A_* = 24.20 mA, *I_B_* = 23.50 mA, injection power *P_jA_* = *P_jB_* = 300 μW.

The effects of external parameter mismatches, such as center wavelength, bias current, and injection power, on the cross-correlation between laser outputs is illustrated in Figure 4. A mismatch of these external parameters for the lasers between Alice and Bob would lead to the reduction of the cross-correlation coefficient. To maintain laser synchronization with cross-correlation exceeding 0.9, the discrepancy ranges for injection power, bias current, and center wavelength are as follows: −0.010~0.004 nm, −7.7~3.4%, and −10~23%, respectively. These tolerated mismatches demonstrate that laser synchronization is less sensitive to external operations and imply synchronization robustness. Nevertheless, a high sensitivity of chaos synchronization to mismatches in laser inner parameters is required to ensure security, which poses difficulties for Eve in obtaining a parameter-matched laser. It has been shown that synchronization is only achievable within a maximum tolerance of ±1% for laser inner parameters, such as active region length, linear gain coefficient, transparency carrier density, and so on [26]. This means that achieving synchronization necessitates the use of lasers manufactured on the same wafer, which is typically unattainable for Eve [33]. It is worth noting that the cross-correlation between two lasers with mismatched internal parameters is significantly lower than the cross-correlation between their outputs and the drive signal. Therefore, extracting information about the key from lasers with mismatched internal parameters is no more effective than extracting it directly from the drive signal.

### 3.2. Secure Key Generation Rate

In this section, we analyze the key generation rate of the scheme based on the previous assumptions. The secret bit rate, which is defined as the ratio of the number of secret bits to the raw samples, can be estimated using the following formula [20]:

(2) $$R_{b}=\frac{1}{M}\left[\left(1-\frac{M_{E}}{M}\right)\left(1-I_{E}\right)-h\left(R_{fail}\right)\right],$$

where *M_E_* is the number of parameter-matched lasers that Eve can manipulate, *I_E_* is the information per bit that Eve knows about Alice or Bob’s retained bits when Eve’s random keying parameter *V_E_* does not match that of Alice and Bob, *R_fail_* is the BER caused by imperfect synchronization between Alice and Bob, and *h*(·) is the binary entropy function defined by *h*(*x*) = −*x*log(*x*) − (1 − *x*)log(1 − *x*). 1/*M* represents the probability of Alice and Bob matching the parameter *V_A_* = *V_B_*, while 1 − *M_E_*/*M* represents the probability for Eve to match a different parameter from Alice and Bob when *V_A_* = *V_B_*. *h*(*R_fail_*) estimates the rate loss caused by the error bits. When the conditions

$$M_{E}<M,\;I_{E}<1,\;h(R_{fail})<\left(1-M_{E}/M\right)\left(1-I_{E}\right),$$

are satisfied, the value of *R_b_* will be positive, indicating that secure key distribution is possible. Next, we will calculate *R_b_* and the key rate based on experimental results and some reasonable assumptions.

If Alice and Bob introduce a binary random keying parameter, the number of possible values of the parameter set is *M* = 2. Considering the sensitivity of the laser’s inner parameters to synchronization, we assume that Eve is unable to obtain a third laser with matching parameters [26], resulting in *M_E_* = 0. Therefore, Eve can only try to extract information from the drive signal. We further assume Eve can obtain bits *X_E,i_* by sampling and quantizing the drive signal using a single threshold [34], and retained those for *i* such that *V_A,i_* = *V_B,i_*. In this case, *I_E_* can be estimated by

(3) $$I_{E}=I\left(Y_{A,B};Y_{E}\right),$$

where *Y_E_* is Eve’s retained bits, *I*(*X*;*Y*) is the mutual information between random bits *X* and *Y*, defined as

(4) $$I\left(X;Y\right)=\sum_{x=0}^{1}\sum_{y=0}^{1}P\left(x,y\right)\log_{2}\frac{P\left(x,y\right)}{P\left(x\right)P\left(y\right)},$$

where *P*(*x*) is the probability of *X* = *x*, *P*(*y*) is the probability of *Y* = *y*, and *P*(*x*,*y*) is the joint probability of *X* = *x* and *Y* = *y*. In practical operation, due to the limited number of available antennas, the drive signal used for estimating *I_E_* is obtained by partitioning a portion of Alice’s received signal.

**Figure 5:**
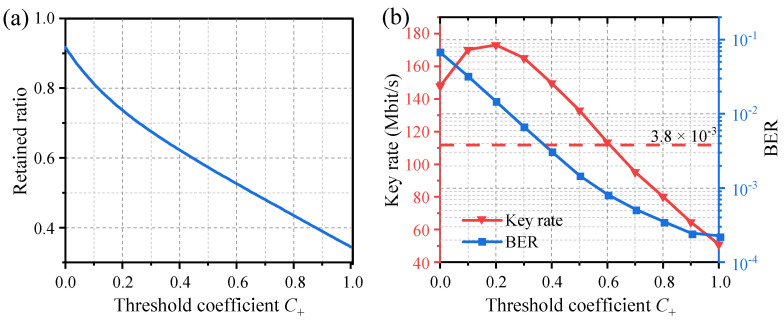
Effects of quantization threshold coefficients on (**a**) the retained ratio, (**b**) the BER between Alice and Bob, and the key generation rate.

A robust dual-threshold quantization method [34] is used to reduce the BER of between Alice and Bob. We set the upper threshold *V_th,u_* and the lower threshold *V_th,l_* as

(5) $$\begin{array}{c} V_{th,u}=m+\sigma C_{+},\\ V_{th,l}=m-\sigma C_{-}, \end{array}$$

where *m* and *σ* are the mean and standard deviation of the temporal waveform, and *C*_+_ and *C*_−_ are the threshold coefficients. A sampling point will be quantized to bit “1” if its voltage is larger than *V_th,u_*, and quantized to bit “0” if its voltage is smaller than *V_th,l_*; otherwise, it will be discarded. To guarantee a nearly uniform distribution of the quantized bit stream, *C*_+_ and *C*_−_ are tuned to satisfy

(6) $$\int_{-\infty }^{V_{th,l}}p[x\left(t\right)]dx(t)=\int_{V_{th,u}}^{+\infty }p[x\left(t\right)]dx(t),$$

where *p*[*x*(*t*)] is the probability density function of the temporal waveform *x*(*t*). In this robust quantization method, only a certain proportion of the sampled points are used to generate the key. The variation of the retained ratio *R_dt_* in relation to the quantization threshold coefficients *C*_+_ is illustrated in Figure 5a. Given this fact, the key rate can be calculated using

(7) $$r=f_{s}R_{dt}R_{b},$$

where *f_s_* is raw sample rate. Considering the bandwidth of the laser output, the raw sampling rate is set to *f_s_* = 1.0 GHz to ensure the randomness of the generated key.

Figure 5b shows the key rate and the BER between Alice and Bob (*R_fail_*) in relation to the quantization threshold coefficients *C*_+_. *R_fail_* decreases as *C*_+_ increases and remains below the BER threshold of 3.8 × 10^−3^ for hard-decision forward-error correction (HD-FEC) when *C*_+_ > 0.4. When *C*_+_ is excessively small or large, the key rate will decrease due to a high BER or low retained ratio. The maximum key generation rate that satisfies the BER threshold is achieved when *C*_+_ = 0.4, resulting in 150 Mbit/s. In this configuration, we summarized statistical measures of secure key generation in Table 1.

Considering the influence of uncertain factors such as noise interference, one can enhance the robustness of the system by increasing the threshold coefficient, at the cost of reducing the key rate. For example, when *C*_+_ = 0.8, the BER decreases to 3.6 × 10^−4^, while the key generation rate decreases to 80 Mbit/s.

**Figure 6:**
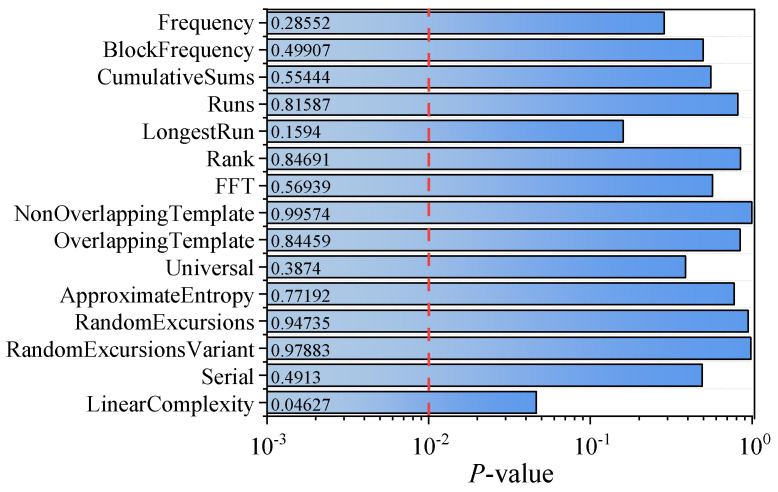
NIST statistical tests results for *p*-value. Using 1 Mbit data and a significant level of α = 0.01, the *p*-value should be greater than 0.01 to pass the test.

In order to verify the randomness of the generated keys, we employed the National Institute of Standards and Technology Special Publication 800-22 statistical tests (NIST SP800-22) to test the generated keys. As shown in Figure 6, the *p*-values are all greater than 0.01, indicating that the generated keys successfully pass all the 15 NIST tests and thereby verify their randomness.

## 4. Numerical Results

We carried out numerical simulations to verify our experimental observations and investigate the impact of transmission distance. For simplicity, we exclusively consider the scenario in free space. The path loss of free space is given by Friis transmission formula [35]

(8) $$L_{f}=20\mathrm{lg}\left(4\pi d/\lambda \right)+L_{M}-G_{t}-G_{r},$$

where *d* is transmission distance, *λ* is the wavelength of the drive signal, *L_M_* is the atmospheric attenuation, and *G_t_* and *G_r_* are the antenna gains of the transmitting and receiving antennas, respectively. The atmospheric attenuation is given by *L_M_* = *γ_a_*·*d*, where *γ_a_* is the attenuation coefficient. Assuming perfect polarization matching between the transmitting and receiving antennas, the transfer function of free space is given by [36]

(9) $$H_{f}\left(f,d\right)=10^{-L_{f}/20}\exp\left(-j2\pi d/\lambda \right),$$

The impulse response of the channel is defined as

(10) $$h_{f}\left(t,d\right)=F^{-1}\;\left\{H_{f}\left(f,d\right)\right\},$$

where *F*^−1^{·} is the inverse Fourier transform. The received signal *S_r_*(*t*, *d*) can be obtained by using

(11) $$S_{r}\left(t,d\right)=S_{t}\left(t\right)⊗h_{f}(t,d)+n\left(t\right),$$

where *S_t_*(*t*) is the drive signal, ⊗ is the convolution operator, and *n*(*t*) is the noise from the receiving antenna output, given by

(12) $$n\left(t\right)=\sqrt{kT_{a}B}\cdot \xi \left(t\right),$$

where *ξ*(*t*) is the normalized Gaussian noise, *k* = 1.38 × 10^−23^ J/K is the Boltzmann’s constant, *T*_a_ is the equivalent noise temperature of the receiving antenna, and *B* is the bandwidth of the receiving antenna. The drive light injected into the DFB laser can be represented by

(13) $$E_{inj}\left(t,d\right)=E_{0}\left(t\right)exp[j\pi g_{a}\cdot S_{r}\left(t,d\right)],$$

where *E*_0_(*t*) is the CW light input to the phase modulator, and *g_a_* is the voltage gain of the amplifier. In our simulations, it’s used the transmission line laser model (TLLM) in VPItransmissionMaker software (Version 9.1) to simulate the dynamic response of DFB lasers to light injection [37,38].

The parameter values used in our simulations are as follows: *G_t_* = 10 dBi, *G_r_* = 10 dBi, *γ_a_* = 0.01 dB/km, *T_a_* = 303.15 K, *B* = 2.8 GHz. The center wavelength of the drive signal is 6.7 GHz, and the bandwidth is 2.8 GHz. The transmission power *P_t_* of the drive signal is 13 dBm, and the modulation depth of the phase modulator is set to 0.7. The laser inner parameters are listed in Table 2. The laser current is set to *I* = 2.4 *I_th_*, where the current threshold *I_th_* is 20 mA. With this configuration, the relaxation oscillation frequency of the laser is 5.5 GHz. The injection strength is set to −8.5 dB, and the optical frequency detuning of the center frequencies of the DFB lasers and drive light is set to 1.6 GHz. We also use a raw sample rate of *f_s_* = 1 GHz and a dual-threshold quantization method to generate bits.

**Figure 7:**
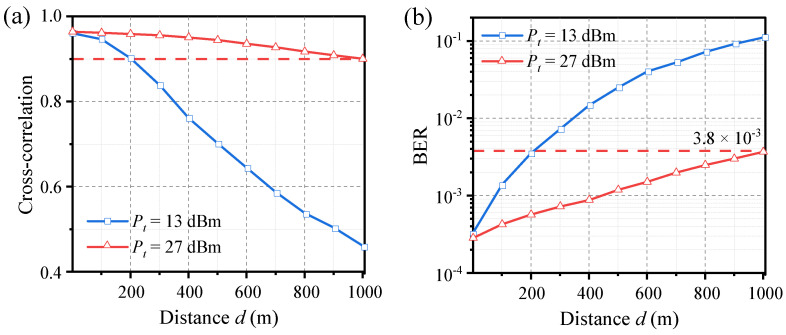
Effects of transmission distance *d* on the (**a**) laser synchronization and (**b**) BER between Alice and Bob. The distance from the drive to Alice and Bob is equal (*d_DA_* = *d_DB_* = *d*). The threshold coefficient of dual-threshold quantization is set to *C*_+_ = 0.8.

First, let us consider the case where the distance from the drive to Alice (*d_DA_*) is equal to the distance from the drive to Bob (*d_DB_*). The simulation results are shown in Figure 7. With the increase in transmission distance *d*, the cross-correlation between the laser outputs decreases, and the BER between Alice and Bob increases. This degradation in performance is due to the decrease in received power as the distance increases, leading to a deterioration of the receiver’s signal-to-noise ratio (SNR), which ultimately affects laser synchronization. When the transmit power of the drive signal is set to *P_t_* = 13 dBm, the maximum transmission distance that satisfies a cross-correlation greater than 0.9 and BER lower than 3.8 × 10^−3^ is approximately 200 m. We can improve the received SNR to extend the maximum distance by increasing the transmit power. When *P_t_* is set to 27 dBm, the maximum distance can reach 1000 m. It is worth noting that the distribution distance between Alice and Bob (*d_AB_*) depends on their orientation relative to the drive. If the drive is positioned exactly in the middle between Alice and Bob, *d_AB_* will be twice the transmission distance.

**Figure 8:**
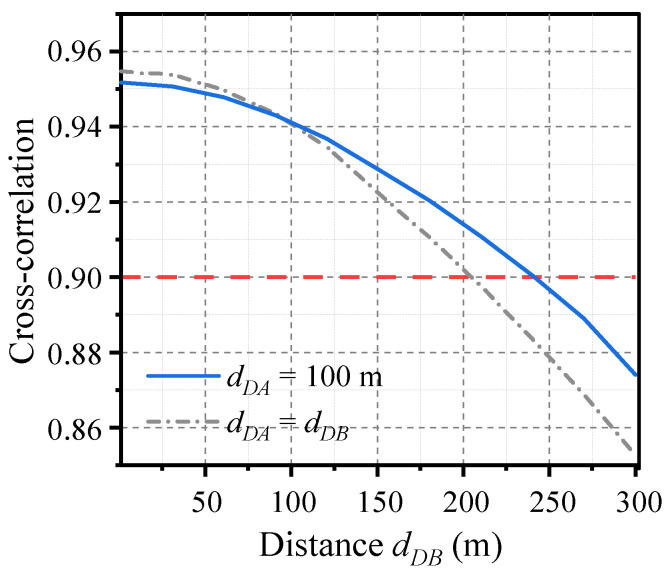
Effect of unequal distances on laser synchronization. The distance from the drive to Alice is fixed at *d_DA_* = 100 m (solid blue curve). As a reference, the results for equal distances from the drive to Alice and Bob (*d_DA_* = *d_DB_*) are also plotted (dashed gray curve). The transmit power is set to *P_t_* = 13 dBm.

Continuing with the more general case where the distance from the drive to Alice and Bob is unequal, the simulation results are shown in Figure 8. The distance from the drive to Bob (*d_DB_*) is adjusted while maintaining the distance from the drive to Alice at *d_DA_* = 100 m. When *d_DB_* is less than 240 m, the cross-correlation of the laser outputs remains above 0.9. This indicates that synchronization exhibits robustness to unequal distances. Therefore, it is also feasible to place the drive at either Alice or Bob, in which case the distribution distance *d_AB_* is equal to the transmission distance.

## 5. Discussion

In this work, we have successfully demonstrated laser synchronization induced by a common wireless signal, achieving a cross-correlation of 0.95. This finding supports the feasibility of common-signal-induced laser synchronization when there is no direct optical connection between the drive source and response laser. It enables us to achieve high-speed key distribution over wireless links utilizing the wideband synchronized output of the laser. Moreover, it is worth noting that the key rate can be further improved by using a wider bandwidth drive signal, thanks to the positive correlation between the laser output and the drive signal’s bandwidth. Nevertheless, the channel bandwidth remains the primary limiting factor in determining the achievable key rate, as it dictates the maximum bandwidth of the drive signal that can be transmitted.

While we utilized a frequency band centered around 6.7 GHz for the drive signal in our experiment, it is not a strict requirement. We observed that when the center frequency of the drive signal is close to the laser’s relaxation oscillation frequency, it is easier to achieve synchronization and obtain a stronger response intensity. Typically, the relaxation oscillation frequency of a laser can be adjusted within the range of 1 to 10 GHz by varying the bias current, allowing for the usability of frequency bands within this range [39].

Our results demonstrate the feasibility of wireless key distribution at high speeds using common-signal-induced laser synchronization. When operating in a free space environment, the method of generating keys based on channel measurements is ineffective. In such cases, our scheme could serve as a valuable alternative, offering a high-speed key distribution rate. Within complex environments, such as multipath fading channels, severe changes in the receiving signal may significantly impair the efficacy of laser synchronization, ultimately leading to a reduction in key rate. By utilizing a digital signal as the drive signal, this difficulty may be resolved in an innovative manner. In combination with the robustness of digital wireless communication and the privacy of laser synchronization, high-speed security key distribution is expected in complex environments, and related work is currently under investigation.

## 6. Conclusions

In this paper, we propose a high-speed wireless key distribution scheme based on the chaos synchronization of nonlinear oscillators and experimentally demonstrate it using DFB lasers. Our experiments demonstrate laser synchronization induced by a common wireless signal with a cross-correlation coefficient of 0.95. Based on the broadband synchronization responses of the lasers, a secure key generation rate of up to 150 Mbit/s with a BER below 3.8 × 10^−3^ is accomplished. Our numerical results show that the distribution distance of the scheme can reach several hundred meters in a free space. The findings of our study indicate that the utilization of common-signal-induced laser synchronization holds promise as a viable approach for attaining high-speed wireless key distribution.

## Figures and Tables

**Table 1:** Statistical evaluation on the generated bits. The threshold coefficients for the dual-threshold quantization are set to *C*_+_ = 0.400 and *C*_−_ = 0.408.

Measure	Symbol	Value
Cross-correlation	*CC*	0.950
Retained ratio by dual-threshold quantization	*R_dt_*	0.623
Mutual information between Alice and Eve	*I*(*Y_A_*,*Y_E_*)	0.489
BER between Alice and Bob	*R_fail_*	3.12 × 10^−3^
Secret bit rate	*R_b_*	0.240
Key generation rate	*r*	150 Mbit/s

**Table 2:** Parameter values of DFB lasers used in simulations.

Parameter	Symbol	Value
Active region length	*L*	350 µm
Active region width	*w*	2.5 µm
Active region thickness	*d*	0.2 µm
Group refractive index	*n_g_*	3.7
Internal loss	*α_0_*	3000 m^−1^
Confinement factor	*Γ*	0.3
Index grating coupling coefficient	*k_i_*	6000 m^−1^
Linear recombination coefficient	*A*	3 × 10^8^ s^−1^
Bimolecular recombination coefficient	*B*	1 × 10^−16^ m^3^s^−1^
Auger recombination coefficient	*C*	1.3 × 10^−41^ m^6^s^−1^
Transparency carrier density	*N_0_*	1.5 × 10^24^ m^−3^
Linear gain coefficient	*g_L_*	3 × 10^−20^ m^2^
Nonlinear gain saturation coefficient	*ε_NL_*	1 × 10^−23^ m^3^
Linewidth enhancement factor	*α_H_*	3
Inversion parameter	*n_sp_*	2

## Data Availability

The data presented in this study are available on request from the corresponding author.
